# SMC3 knockdown triggers genomic instability and p53-dependent apoptosis in human and zebrafish cells

**DOI:** 10.1186/1476-4598-5-52

**Published:** 2006-11-02

**Authors:** Giancarlo Ghiselli

**Affiliations:** 1 Department of Pathology and Cell Biology, Thomas Jefferson University, 1020 Locust Street, Philadelphia, PA 19107, USA

## Abstract

**Background:**

The structural maintenance of chromosome 3 (SMC3) protein is a constituent of a number of nuclear multimeric protein complexes that are involved in DNA recombination and repair in addition to chromosomal segregation. Overexpression of SMC3 activates a tumorigenic cascade through which mammalian cells acquire a transformed phenotype. This has led us to examine in depth how SMC3 level affects cell growth and genomic stability. In this paper the effect of SMC3 knockdown has been investigated.

**Results:**

Mammalian cells that are SMC3 deficient fail to expand in a clonal population. In order to shed light on the underlying mechanism, experiments were conducted in zebrafish embryos in which cell competence to undergo apoptosis is acquired at specific stages of development and affects tissue morphogenesis. Zebrafish Smc3 is 95% identical to the human protein, is maternally contributed, and is expressed ubiquitously at all developmental stages. Antisense-mediated loss of Smc3 function leads to increased apoptosis in Smc3 expressing cells of the developing tail and notocord causing morphological malformations. The apoptosis and the ensuing phenotype can be suppressed by injection of a p53-specific MO that blocks the generation of endogenous p53 protein. Results in human cells constitutively lacking p53 or BAX, confirmed that a p53-dependent pathway mediates apoptosis in SMC3-deficient cells. A population of aneuploid cells accumulated in zebrafish embryos following Smc3-knockdown whereas in human cells the transient downregulation of SMC3 level lead to the generation of cells with amplified centrosome number.

**Conclusion:**

Smc3 is required for normal embryonic development. Its deficiency affects the morphogenesis of tissues with high mitotic index by triggering an apoptotic cascade involving p53 and the downstream p53 target gene *bax*. Cells with low SMC3 level display centrosome abnormalities that can lead to or are the consequence of dysfunctional mitosis and/or aneuploidy. Collectively the data support the view that SMC3 deficiency affects chromosomal stability leading to the activation of p53-dependent mitotic checkpoint.

## Background

The structural maintenance of chromosome 3 protein (SMC3) has been first identified as a key component of the multimeric protein complex cohesin that plays an essential role during the segregation of sister chromatids [1-3]. In addition to chromosomal segregation, SMC3 is also involved in DNA recombination and repair [4]. A multimeric complex containing BRCA1 in addition to SMC3 plays a key role as effectors of the ATM/NBS1 DNA-damage surveillance pathway [5,6]. Recently SMC3, like p53 and BRCA1, has been identified as the target of the serine/threonine kinase Chk2 that plays a critical role in the DNA damage checkpoint pathway [7]. Given its involvement in pathways affecting genomic stability, SMC3 level alteration is likely to have a significant impact on the cellular genetic integrity. Consistent with this view, elevated SMC3 level has been detected in a significant (~ 60%) fraction of human colon carcinoma and in the tumoral areas of mice genetically prone to develop polyps [8,9]. In mammalian cells the ectopic expression of SMC3 triggers their transformation to a growth attachment-independent phenotype [8]. Furthermore SMC3 upregulation affects the expression of members of the ras-rho/GTPase and CREB oncogenic pathways that are key players in cell cycle regulation, microtubule dynamics, and in cell differentiation and survival [10].

Pioneering work in yeast mutants have shown that Smc3 deficiency leads to the premature separation of sister chromatids [11] and the disruption of the mitotic process. However key differences exist in the regulation of sister chromatid cohesion between yeast and higher eukaryotic cells. In particular the timing of dissociation of the cohesin complex from chromatin at the onset of anaphase is different [12,13]. How cells of vertebrates respond to SMC3 downregulation has not been examined in detail. Low expression of SMC3 has been detected in rat kidney epithelial cells transfected with Ha-ras [14], in lung epithelial cells infected with WSN virus [15], and in damaged axons [16], suggesting that downregulation of SMC3 is part of the response to oncogenes and stress. Knockdown of Smc3 in *c.elegans *affects chromosome segregation during mitosis and is embryonically lethal, but the underlying mechanism has not been investigated [17]. Other studies have shown that interference with the formation or dissolution of the cohesin complex causes abnormal mitosis [18]. Knockdown of either the cystein protease securin or of its substrate separase, two key components of the machinery that free sister chromatids from the grip of cohesin at the onset of anaphase, leads to aneuploidy [19,20]. Since SMC3 is an essential component of the cohesin complex, its deficiency conceivably affects the complex formation or its function [21]. The study of the biology of SMC3 deficiency is however hampered by the fact that mammalian cells that are SMC3-deficient fail to develop into a clonal population and are selectively eliminated. To examine the mechanism and identify the chain of events, we have turned to zebrafish (*danio rerio*), a developmental model where apoptosis and cell growth arrest can be easily investigated in the whole organism [22,23]. Zebrafish utilizes cell cycle arrest and apoptosis (p53-dependent and independent) to control morphogenesis [24]. The ability to initiate cell cycle arrest and apoptosis is however acquired at different time during development [24-26] thus offering the opportunity of discriminating the effect of a gene product on each pathways. We have discovered that in zebrafish SMC3 deficiency triggers apoptosis in highly proliferative areas such as those in the developing central nervous system and the tail. We have furthermore established that the morphological changes occurring in Smc3-deficient embryos are secondary to p53-mediated apoptosis that is triggered by the activation of the mitotic checkpoint.

## Results

### Cloning and chromosomal mapping of zebrafish *smc3*

**Figure 1:**
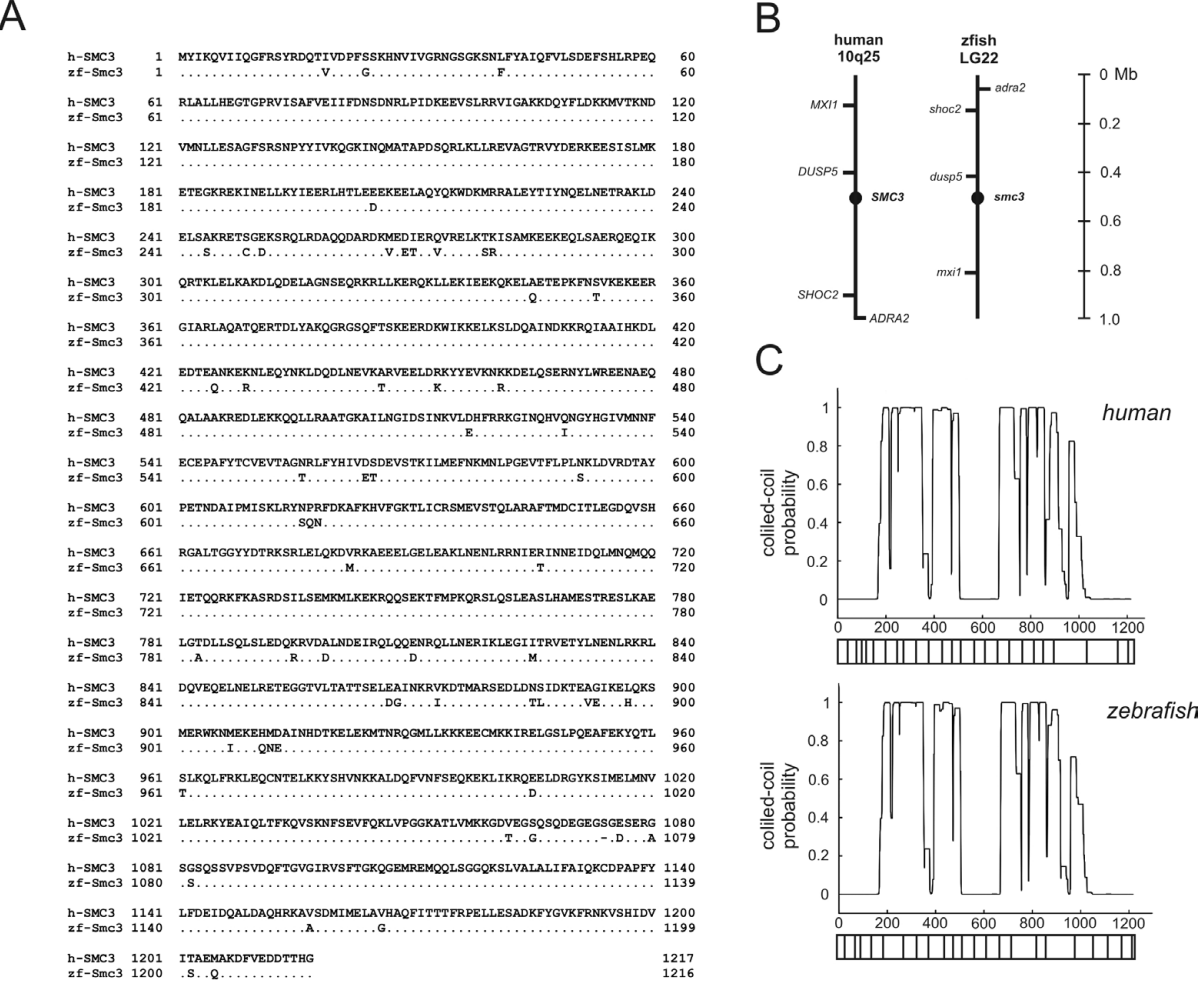
***Cloning and chromosomal mapping of the zebrafish smc3 gene***. A) The ORF of the zebrafish *smc3 *gene was assembled by aligning the sequences of the EST clones identified through a search of the zebrafish genome and dEST databases using the human SMC3 polypeptide sequence as query. Zebrafish *smc3 *encodes a protein that is >95% identical to the human protein. B) The zebrafish *smc3 *locus is synthenic to the human *SMC3 *locus. Genes mapping in close proximity to both the *smc3 *and *SMC3 *gene locus are also identified. *MXI*: max interactor protein 1 (a MYC-binding protein); *DUSP5*: dual specificity phosophatase 5; *SHOC2*: suppressor of *clear *homolog (a RAS-binding protein); *ADRA2*: alpha2A adrenergic receptor. C) Prediction of the protein coiled-coil domains. The numbers on the abscissa corresponds to the amino acid residues. The probability of the polypeptide to assume a coiled coil conformation is plotted on the ordinate axis. The contribution of the gene's exons to the five-domain structural organization of the protein is illustrated at the bottom of the coiled-coil probability plot.

In order to identify and characterize the zebrafish *smc3 *gene, the coding sequence of the human gene was used to query the NCBI zebrafish genome and dEST databases. The alignment of the sequences retrieved enabled to derive the putative sequence of the zebrafish Smc3 protein (Genbank BC044408). Smc3 is a polypeptide of 1216 amino acids that is >95% identical to the human protein (Figure 1A). Data mining of the genomic database showed that Smc3 is encoded by 23 exons harbored in a genomic region of 48 kb. In dEST and genomic databases screens, no other zebrafish genes with higher similarity to other subclasses of the cohesin family than to Smc3, was found. Thus, like other vertebrates, including humans, zebrafish has a single *smc3 *gene. The zebrafish *smc3 *locus is located on chromosome 22 between markers Z14657 and Z66142. Based on the mapping of neighbor genes, this chromosomal region is synthenic with human chromosome 10q25, i. e. the region harboring the *SMC3 *gene (Figure 1B). The analysis of the protein secondary structure utilizing the COIL algorithms revealed that the conformational structure of Smc3 is fully conserved in the human protein (Figure 1C). In particular the location of the coiled-coil domains and the breaks in the coiled conformation are fully conserved thus corroborating the idea that these are key structural features that are necessary for the conserved biological functions of SMC3 [27].

### Smc3 is maternally and zygotically derived

**Figure 2:**
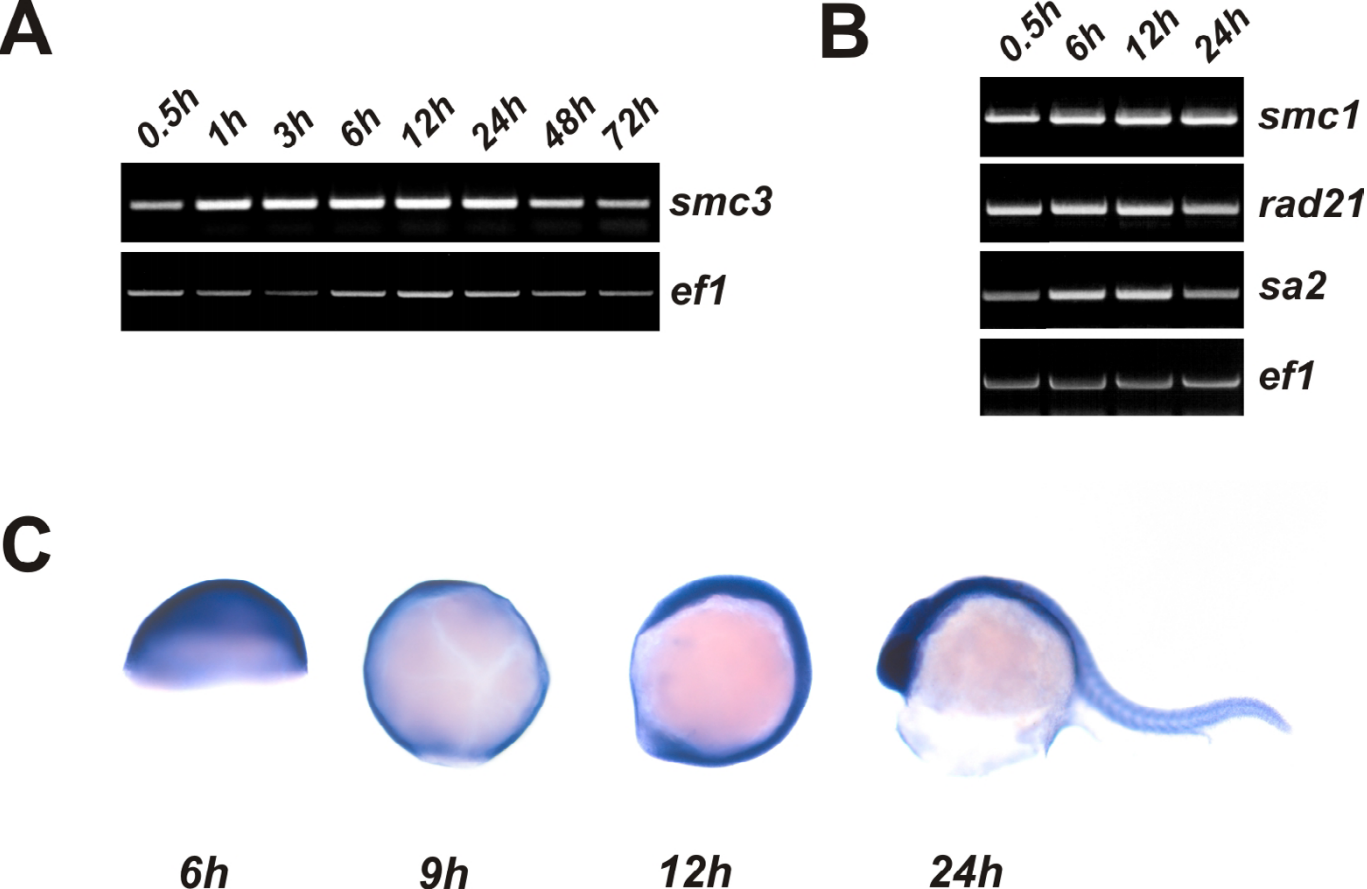
***Expression of zebrafish smc3 during embryogenesis***. A) Semiquantitative RT-PCR analysis of *smc3 *transcript level in zebrafish embryos at different times during development. *ef-1 *transcript level, which is expressed throughout development, was used as internal control. B) Expression of constituents of the cohesin multimeric protein complex: zebrafish *smc1*, *rad21 *and *sa2 *transcripts were quantitated as described in panel A. C) Spatio-temporal expression of *smc3 *mRNA. Whole-mount mRNA *in-situ *hybridization was performed at the indicated developmental stages. Left-to-right: lateral view with the animal pole up of embryos at 50% epiboly (6 hpf), at 90% epiboly (9 hpf), at 3-somite stage (12 hpf), and at 24 hpf.

The temporal expression pattern of *smc3 *during zebrafish development was investigated by semiquantitative RT-PCR. *smc3 *transcript was detected in fertilized embryos at developmental stages prior to the onset of zygotic transcription, indicating that this message is maternally derived. *smc3 *transcript level remained elevated until the end of the segmentation stage (Figure 2A) but decreased thereafter. The expression of *smc1*, *rad21 *and *sa2*, whose products together with that of *smc3 *are components the cohesin protein complex, was detected in embryos since the early cell divisions (Figure 2B) thus supporting the conclusion that a functional cohesin complex is operational since the very early stages of embryogenesis. *smc3 *transcript localization in the embryo at different developmental stages was investigated by whole-mount *in-situ *hybridization using gene-specific antisense riboprobes (Figure 2C). Throughout the early development stages up to 9 hpf a diffuse staining was observed throughout the blastoderm. At the beginning of the segmentation stage the staining was detected along the entire dorsal axis. At 24 hpf *smc3 *expression was higher in the head region and was weaker in the tail. Staining specificity was assessed using a sense riboprobe in which case no staining was observed (data not shown).

### Smc3 deficiency causes cell death affecting embryonic development

**Figure 3:**
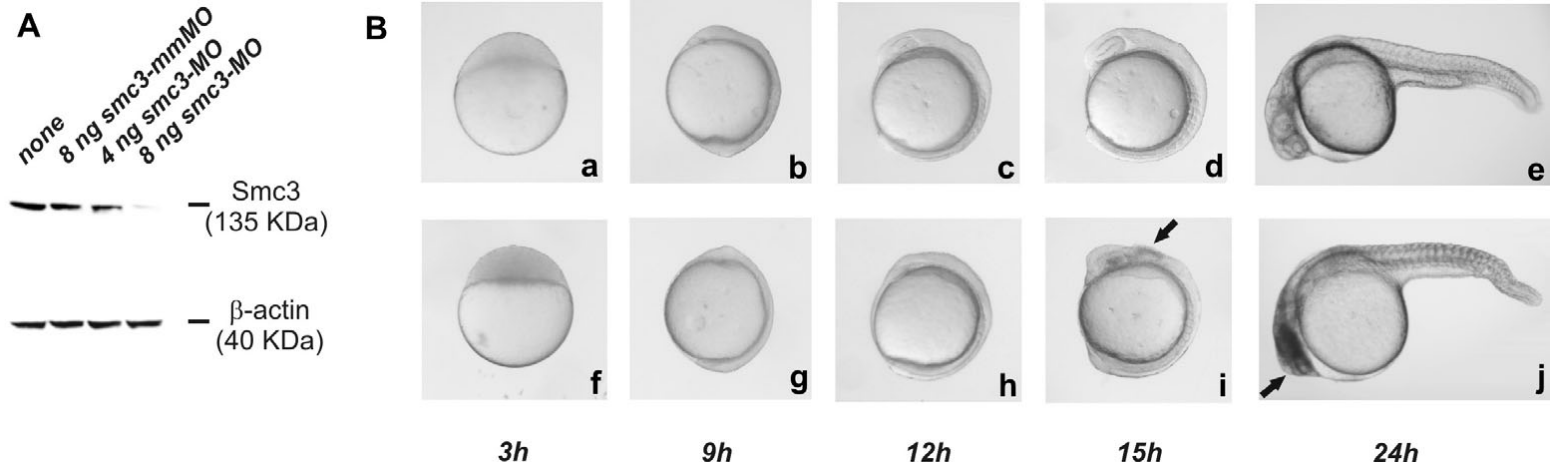
***Effect of Smc3 knockdown on embryogenesis***. A) Western immunoblot analysis of Smc3 level in embryos injected at 1–2 cell stage with increasing doses of *smc3*-MO or *smc3*-mmMO. At 24 hpf 10 embryos were dechorionated and the yolk remnant removed. After homogenization in 100 μl lysis buffer, proteins were separated on 7.5% SDS-PAGE and Smc3 detected by immunoblotting using goat anti-human SMC3 antibody followed by ECL reaction. B) Morphogenesis in normal (a-e) and morphant (f-j) embryos injected with 8 ng of *smc3*-MO. At the indicated times embryos were collected and their morphology examined. Note the presence of necrotic cells in the eye and the cerebellar region of embryos at 15 hpf. At 24 hpf the majority of the morphants display head necrosis and a range of tail developmental defects including a reduction of the tail extension, the loss of chevron-like structure of the somites and the narrowing or disappearance of the notocord.

Attempts to generate SMC3-deficient clones of mammalian cells has lead us to believe that mammalian cells tolerate a marginal decrease in SMC3 level beyond which apoptosis is triggered (this lab's unpublished observations, 2006). To investigate whether SMC3 deficiency has a similar pro-apoptotic effect in a complex cellular context, zebrafish embryos were utilized as model system. The components of the p53-dependent apoptotic pathway of mammalian cells are conserved in the zebrafish embryo and the pathway is activated at specific times during development [22,24]. Apoptotic cells can easily be identified owing to the optical transparency of the embryo and since zebrafish utilizes apoptosis to direct morphogenesis, the process can be monitored in real time. In addition techniques have been developed that allow to modulate the level of any given zebrafish gene product [28]. To achieve Smc3 knockdown, zebrafish were injected at the one-to-two cell stage with *smc3*-targeting morpholino-modified antisense oligonuclotides (MO) to block *smc3 *mRNA translation. In order to assess the efficacy and the specificity of the antisense oligos, MO targeting the 5'-UT region of the *smc3 *gene (*smc3*-MO) and a 4-bases mismatch control oligo (*smc3*-mmMO) were injected at increasing doses in the embryos' yolk. The administration of *smc3*-MO (1–8 ng/embryo) leads to a dose-dependent knockdown of Smc3 protein in embryos at 24 hpf as revealed by Western immunoblot analysis of the whole embryo lysate (Figure 3A). On the other hand, injection of the control *smc3*-mmMO (8 ng/embryo) has no effect. Embryo injected with *smc3*-MO did not display significant morphological alteration until somitogenesis (~ 12 hpf) (Figure 3B). At five-somite stage, necrosis was however evident in the eye and in the cerebellar region. At 24 hpf, in addition to brain necrosis, the morphants displayed an overall reduction of the body length. In particular the tail region was not properly extend. The tail fin was absent and the terminal region of the tail was collapsed. In addition the notocord morphology was significantly affected since unlike the characteristic tight cell packaging usually observed at this developmental stage, cells appeared round and disorganized. The severity of the morphological changes was a function of the dose of MO administered (Table 1). A second MO targeting a different sequence of the 5'-UT region of *smc3 *induced the same morphological changes. The *smc3 *morphants could be rescued by the ectopic expression of Smc3 by administering capped *smc3 *mRNA confirming the specificity of the biological response observed (Table 1).

**Table 1:** Effect of SMC3-deficiency on zebrafish development.

**Treatments**	**Embryos no.**	**Apoptotic Phenotype (%)**
*(A) Uninjected*		
*1 nl Danieau buffer*	60	0%
		
*(B) SMC3 Morpholino-antisense*		
*smc3-MO *(1 ng)	60	45%
*smc3-MO *(2 ng)	60	56%
*smc3-MO *(4 ng)	60	86%
*smc3-MO *(8 ng)	60	100%
*smc3-mmMO *(8 ng)	60	5%
		
*(C) zfSMC3 rescue*		
*smc3-MO *(4 ng)/*smc3 mRNA *(250 pg)	60	15%
		
*(D) p53 Morpholino-antisense rescue*		
*p53-MO *(4 ng)	60	0%
*smc3-MO *(4 ng)/*p53-MO *(4 ng)	60	0%

Embryos at 1–2 cell stage were injected in the yolk with 1–3 nl of MO or mRNA to achieve the indicated dose. Each injection session consisted of 2–3 treatment groups of 30 embryos each and several experiments were performed to reach the sample size indicated. Embryos serving as the untreated group were injected with 1 nl of Danieau buffer. Embryos receiving a dual treatment, were first received smc3-MO and after randomization, half was injected with p53-MO or smc3-RNA. At 6 hpf the unfertilized eggs were removed and the developing embryos analyzed at 15 hpf. Embryos showing necrosis in the head were classified as apoptotic.

### Smc3 deficiency activates a p53-dependent pathway

**Figure 4:**
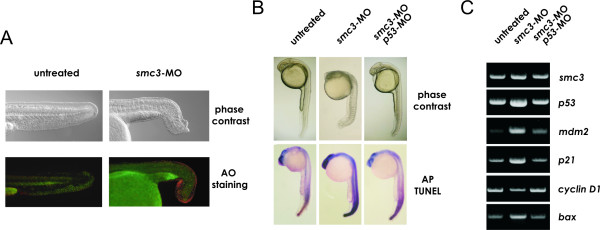
***p53 pathway activation and cell death following Smc3 knockdown***. A) Acrydine Orange (AO) staining of apoptotic cells. Embryos at 1-2-two cell stage were injected with 1 nl of Danieau buffer (untreated group) or with 8 ng/embryo *smc3*-MO and at 24 hpf incubated in E3 medium containing 5 μg/ml of the fluorescent dye. Following 1 h incubation, the embryos were washed in PBS and observed under a fluorescence microscope. Note the high level of fluorescence signal at the terminal edge of the morphant's tail whereas in the untreated embryo fluorescence is only detected at the prospective vent. B) Morphological assessment and TUNEL immunostaining of apoptotic and dead cells in morphants. Embryos were injected at 1–2 cell-stage with MO to block translation of either *smc3 *only or of *smc3 *and *p53*, and examined at 28 hpf. C) Semi-quantitative RT-PCR of *smc3*, *p53 *and a p53-target genes transcript level in untreated embryos and in morphants at 24 hpf

**Figure 5:**
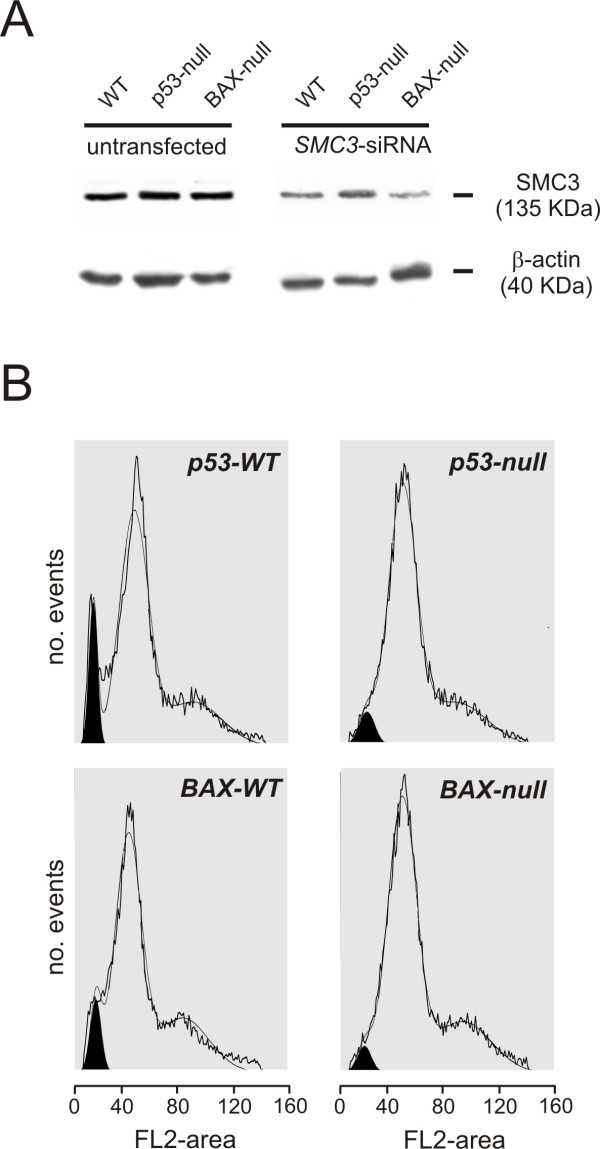
***Role of p53 and BAX in SMC3-deficiency mediated apoptosis in human HCT116 cells***. A) Western immunoblot assay of SMC3. p53-null, BAX-null and the matching wild-type parental HCT116 cells were transfected with 50 ng/ml *SMC3*-siRNA (5'-AACAGCGGUGGCUUUGC-3') and harvested 72 h later in lysis buffer. Proteins were separated on 7.5% SDS-PAGE. After transfer onto a nitrocellulose filter, SMC3 was detected using a goat anti-SMC3 antibody followed by ECL reaction. For normalization of the protein loading, the filters were stripped of the SMC3 immunocomplexes and reacted with anti-human β-actin antibody. Semiquantitative analysis of the protein level was performed by densitometric scanning of the autoradiographs. B) Flow cytometry of the cell DNA content. At 72 h post-transfection, cells were collected by trypsinization and fixed overnight in 70% ethanol at 4°C. After centrifugation, cells were washed once in PBS and then incubated with propidium iodide/RNase. Cell DNA fluorescence intensity was analyzed on a Coulter Epic flow cytometer. Results were gated and deconvoluted with ModFitLT to determine the fraction of apoptotic cells with < 2 N DNA fluorescence intensity. In the graphs the fraction of apoptotic cells is highlighted in black.

Experiments were performed to examine the cellular abnormalities underlying the altered morphology of the affected tissues. When stained *in vivo *with Acridine Orange, a membrane-permeable aromatic derivative that preferentially stains apoptotic cells, 24 hpf Smc3-deficient embryos displayed intense signal at the outer perimeter of the tail tip (Figure 4A). By contrast in the untreated embryos of the same age, apoptosis was confined to the prospective vent. In previous studies apoptosis in zebrafish during early development has been shown to be associated with activation of p53-dependent pathways [22,29-31]. In order to examine the role of p53 in mediating cell death in Smc3-deficient embryos, experiments were conducted in a p53-deficient background. TUNEL assay performed on the whole embryo allowed to examine the spatial distribution of apoptotic/necrotic cells at 28 hpf. The results revealed a significant increase in the number of apoptotic cells in the brain and the tail of embryos following Smc3 knockdown but not when *p53*-MO was co-administered with *smc3*-MO (Figure 4B). To investigate downstream p53-dependent transcriptional responses in the zebrafish, we examined the expression of p53 target genes such as *p21*^*WAF*1/*CIP*1 ^and *cyclin D1*, based on their central role in cell-cycle arrest; *bax*, for its involvement in the apoptosis pathway; and *p53 *and *mdm2 *to explore the feedback loops downstream of p53 (Figure 4C). In 24 hpf Smc3-deficient embryos semiquantitative RT-PCR analysis showed that p53 transcript level was elevated approximately two-folds. In addition p53 protein activity was enhanced as indicated by the two fold increase in mRNA levels of the target genes *mdm2*, *p21*^*WAF*1/*CIP*1^, and *bax*. At the same time *cyclin D1 *expression was decreased suggesting a cell cycle delay at the G1-S transition. Conversely in a p53-null background, Smc3 knockdown did not affect the expression of the p53-target genes. These finding prompted us to investigate whether p53 is required for apoptosis in SMC3-deficient mammalian cells. In these experiments somatic human cells constitutively lacking either p53 or BAX and their wild type parental cell lines, were utilized. To achieve a status of SMC3 deficiency cells were transfected with *SMC3*-specific siRNA oligonucleotides and harvested 72 h later. Western immunoblot analysis showed that SMC3 protein level in the transfected cells had been decreased by 70% or more (Figure 5A). Interestingly the knockdown of SMC3 had virtually no effect on the level of the SMC1 (see Additional Figure 1) further suggesting that the concentration of the two proteins is regulated independently [10]. In p53 proficient (wild-type) HCT116 cells, SMC3 deficiency triggered significant apoptosis as evidenced by FACS analysis of the cell DNA content (Figure 5B). On the other hand, SMC3 deficiency failed to trigger apoptosis in HCT116 cells lacking p53. Further corroborating the idea that a p53-dependent apoptotic pathway is responsible for cell death following SMC3 knockdown, cells lacking BAX, the terminal mediator of the p53-dependent apoptotic cascade, were refractory to apoptosis after treatment with *SMC3*-siRNA.

### Smc3-deficiency triggers genomic instability

**Figure 6:**
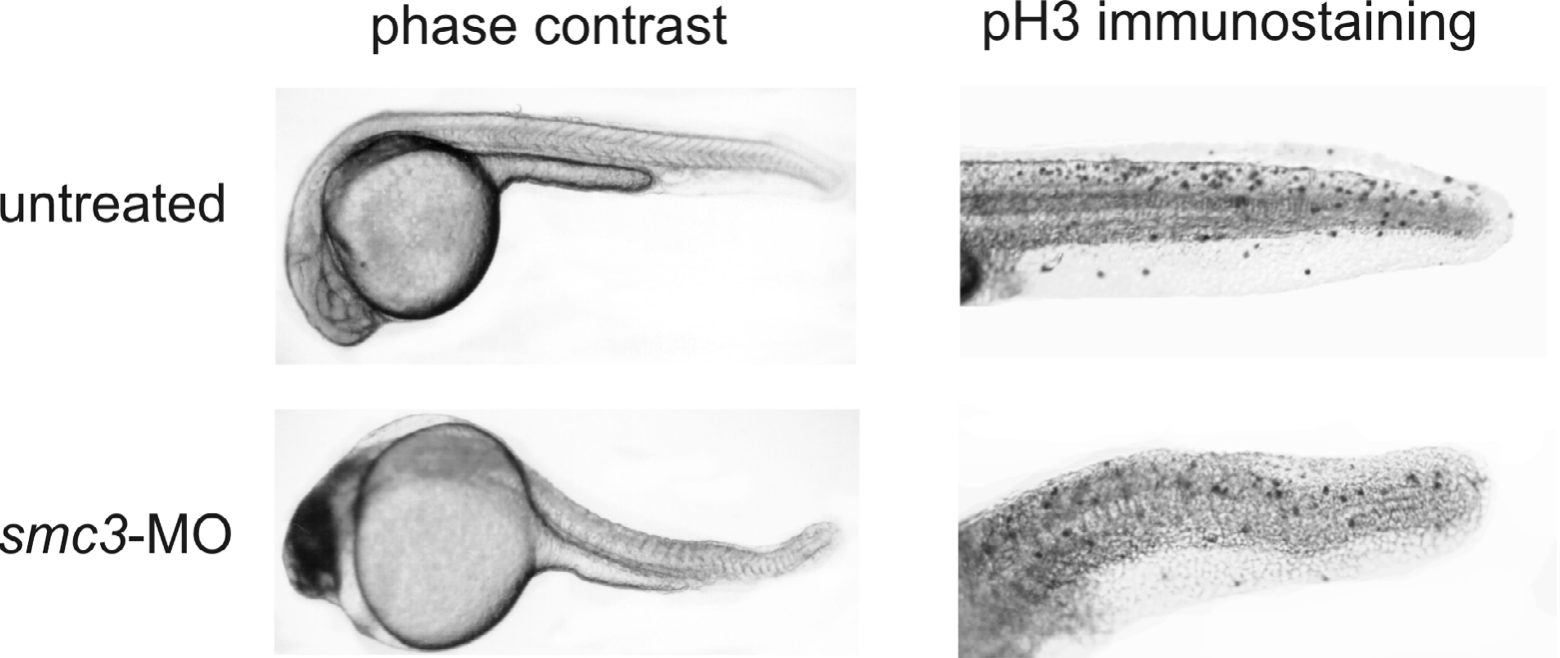
***Effect of Smc3 knockdown on the phosphorylation in vivo of histone H3***. Embryos at 1-2-two cell stage were injected with the vehicle alone or with 8 ng/embryo *smc3*-MO and analyzed at 24 hpf. Embryos were fixed overnight in 4% para-formaldehyde and permeabilized by immersion in -20°C acetone for 10 min. After overnight incubation at 4°C with 1 μg/ml rabbit anti-pH3 antibody, the embryos were placed in 1 μg/ml HRP-conjugated goat anti-rabbit IgG solution and the immunocomplexes detected by reaction with DAB reagent. The punctuate staining pattern visible in the embryo tails identifies the localization of the pH3 antigen and thus of the mitotic cells.

**Figure 7:**
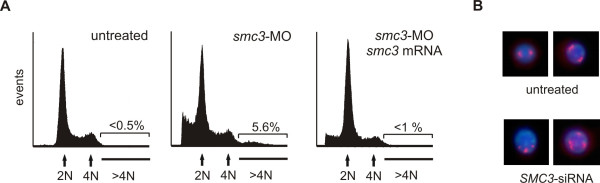
***Cell DNA content analysis and chromosomal abnormalities following SMC3 knockdown***. A) Cell cycle FACS analysis of zebrafish embryonic cells. Embryos at 1–2 cell stage were injected with 1 nl Danieau buffer (left-hand side) or 4 ng of *smc3*-MO (center) or 4 ng of *smc3*-MO together with 250 pg *smc3 *mRNA (right-hand side). Batches of 30 embryos at 24 hpf were incubated in 0.05% trypsin 15 min at 25°C and the cells dissociated by pipetting through a large bore glass pipette. After fixation in 70% ethanol at 4°C overnight, cells were stained with propidium iodine/RNase, and the DNA cell content analyzed by flow cytometry. Gated data were used for analysis. Note the presence of a population of aneuploid (DNA > 4) cells in embryos following Smc3 knockdown. B) Analysis of the centrosome organization in human cells. Human 293 cells were transfected with 50 ng/ml of *SMC3*-siRNA and after 24 h plated on a poly-lysine-coated coverslip. Fourty-eight h later the cells were fixed in 4% para-formaldehyde, permebilized and then incubated with 1:1000 anti-human γ-tubulin antibody. Following incubation with Alexafluor 567-conjugated anti-rabbit IgG (Molecular Probes) and DNA staining with DAPI, the cells were examined under a fluorescence/UV microscope and the red (Alexafluor 567) and blue (DAPI) signals combined.

p53 functions primarily as a transducer of DNA damage signals in cells of higher eukaryotes. Upon DNA damage or other cellular stress, p53 becomes activated and induces either cell cycle arrest to allow DNA repair, or apoptosis to eliminate cells with elevated carcinogenic potential. In order to distinguish between these two possible outcomes, we assessed the number of mitotic cells by performing embryo whole-mount immunostaining of phosphohistone H3 (pH3), a marker for cells that have entered mitosis (Figure 6). Thanks to the embryo transparency pH3-reactive cells could be detected throughout the animal body. At 24 hpf mitotic cells were encountered at high frequency in the tail (105 ± 18 pH3-positive cells/tail, n = 3). Although in lower number than that seen in the untreated embryos, several mitotic cells were also detected in the tail of Smc3-deficient embryos (73 ± 12 cells/tail, n = 3), supporting the conclusion that increased apoptosis as opposed to a block of cell division is mainly responsible for the morphological alteration in embryos following Smc3 knockdown. This finding is consistent with the results of previous studies [24,32] that had concluded that after midgastrula stage, the zebrafish embryos employs apoptosis, in preference to cell proliferation arrest, to eliminate cells with damaged genetic content. Given the role that SMC3 plays during mitosis in all eukaryotic cells, abnormalities in chromosomal segregation are expected in SMC3-deficient vertebrate cells. The presence of abnormal chromosomes would then lead to the activation of both p53-dependent and independent mitotic checkpoints triggering the apoptotic cascade through which cells with aberrant chromosomes content are eliminated. In order to corroborate this hypothesis we sought for evidences of abnormal mitosis in SMC3-deficient zebrafish and human cells. During early zebrafish embryogenesis, cells divide every 30 min for the first 5.4 h [33,34]. It is estimated that upon reaching the end of the segmentation stage zebrafish somatic cells have undergone no less than a dozen cell divisions. Although cells that have undergone defective mitosis are efficiently eliminated through p53-mediated apoptosis [24,26], if their number is large some may escape elimination and a population of aneuploid cells may thus become evident. In agreement with this scenario along with a high proportion of apoptotic/necrotic cells, a significant fraction of aneuploid cells with larger then normal DNA content were detected by FACS analysis in Smc3 deficient embryo (Figure 7A). Unlike the zebrafish embryonic cells, human cells duplicate at far lower rate and FACS analysis is not suitable to detect the small number of aneuploid cells generated after few mitosis. However defects in mitosis can be identified in mammalian cells before they enter programmed cell death by monitoring the number of centrosomes. At the onset of mitosis, normal cells contain two centrosomes that are located at the poles of the mitotic spindle. On the other hand a larger number of centrosomes is seen in those cells that have undergone defective mitosis [35]. Centrosome number abnormalities may be either the cause or the consequence of abnormal mitosis [36]. Immunostaining of γ-tubulin – a highly conserved constituent of this organelle, revealed that the centrosome organization is disrupted in a significant fraction of human 293 cells with low SMC3 level (Figure 7B). The analysis performed on 200 cells per group revealed that one-fifth (43/200) of the SMC3-deficient cells display evidence of multipolar spindles whereas only few (3/200) of the normal cells display this defect.

## Discussion

Cell death is the driving force in the morphogenesis of developing tissues as well as a major factor in homeostasis of adult organs and tissues. The zebrafish embryo is a good model for studying the interplay between genetic and environmental influences on apoptosis [37]. Development is rapid, and embryos remain transparent throughout most of embryogenesis, simplifying the staging and analysis of whole-mounts. Before mid-blastula transition, an "embryonic" type cell cycle is operative, which is characterized by a rapid alternation between S and M phases and an apparent lack of checkpoints to monitor completion of these phases [33]. It has been hypothesized [38] that embryos acquire a general capability for apoptosis as a consequence of passage through the maternal-to-zygotic transition, that is after the maternal determinants (mRNAs and proteins) have been exhausted or degraded and replaced by zygotic determinants. The acquisition of apoptosis capability allows the embryo to eliminate defective cells, which were inadvertently generated during the cleavage phase of development to prevent their contribution to critical lineages later in development. Block of cell growth is an alternative mechanism during embryogenesis that is activated in response to DNA damage. This mechanism operates before embryos become capable of disposing cells through apoptosis [32]. The fact that Smc3-deficient embryos do not display significant growth retardation at any stage of development suggests that cell cycle block is not the preferred mechanism to limit the number of defective cells arising from defective mitosis. This conclusion is also supported by the evidence that in *smc3 *morphants the number of pH3-reactive cells, i. e. of those cells that have entered mitosis, is not significantly affected. Collectively these data thus support the conclusion that apoptosis is specifically triggered when Smc3 level falls. Findings similar to those in the *smc3 *morphants have been recently reported in the *crash&burn *(*crb*) zebrafish mutant that is genetically lacking functional B-myb [39]. Zebrafish B-myb, like the human homolog, plays a role in the cell cycle regulation, particularly at the G2/M transition and the maintenance of normal ploidy. Metaphase spreads demonstrate that *bmyb *mutants contain polyploid and hypertetraploid cells. As the *smc3 *morphants, B-myb-null embryos progress through morphogenesis at normal pace but develop massive apoptosis in the brain and the tail region. These findings suggest that in zebrafish mitotic abnormalities are preferentially dealt with through an apoptotic response. Interestingly haploinsufficiency of *bmyb *leads to an increased rate of tumors after carcinogenesis whereas overexpression data from human tumors suggest that B-myb acts as an oncogene. This observation and others have lead to propose that genes controlling the mitotic checkpoint may affect tumor formation through either inactivation or overexpression [39,40].

In addition to mounting an apoptotic response, cells that are SMC3 or B-myb deficient display a further striking similarity in their centrosomal organization. During mitosis, two centrosomes form spindle poles and direct the formation of bipolar mitotic spindles, which is an essential event for accurate chromosome segregation into daughter cells. The presence of more than two centrosomes severely disturbs mitotic process and cytokinesis via formation of more than two spindle poles, resulting in an increased frequency of chromosome segregation errors [35]. Centrosome amplification is an early event in tumor formation [41,42]. It occurs in almost all type of cancer [35] and is considered as a major contributing factor for chromosome instability in cancer cells leading to aneuploidy [43]. Given that the centrosome organization is disrupted in a significant fraction of cells with low SMC3 level and that the effect is detectable after transient SMC3 level alteration within the context of few cell duplications, we speculate that SMC3 may directly affect the centrosome duplication process. The mechanisms leading to centrosome amplification are still poorly understood. High dose of gamma irradiation that induce DNA strand breaks, induces centrosome amplification but the mechanism through which DNA damage leads to centrosome amplification remains to be elucidated [44]. Conceivably the mechanism involves proteins that like SMC3 are members of the DNA recombination/repair mechanism and of the mitotic spindle checkpoint. Various proteins that associate with centrosomes such as pericentrin, STK15/BTAK/Aurora-A kinase, ATR, BRCA1, BRCA2, have been shown to influence centrosome duplication in human cancer [36,45-49]. Of these proteins, BRCA1 has been shown to bind to SMC3 [5,6]. In addition SMC3 binds to dynactin (this lab's unpublished observation) and KIF3B kinesin [50], two key components of the centrosome multimeric protein complex. The ability of SMC3 to interact with a number of centrosome proteins hints to a possible mechanism through which SMC3 may influence centrosome duplication. Further work is needed to elucidate the potential role of SMC3 on centrosome dynamics that may be also indirect and complex.

## Conclusion

In this paper we have described the cloning of zebrafish *smc3 *and determined that its expression is required for normal embryonic development. Smc3 deficiency triggers an apoptotic cascade involving p53 and the downstream p53 target gene *bax*. The morphology of tissues such as the tail that during normal development utilize apoptosis for remodeling, is drastically altered. The effect of SMC3 deficiency in human cells recapitulates the results in zebrafish and confirm the involvement of p53. Human SMC3-deficient cells contain an excess number of centrosomes, whereas a population of aneuploid cells is detected in *smc3 *morphants suggesting that SMC3 is a gatekeeper of genetic stability. We postulate that the deviation of SMC3 from a certain level leads to catastrophic mitoses. The ensuing aneuploidy in turn activates the p53 apoptotic pathway leading to the observed morphological changes. Because aneuploidy is thought to contribute to malignant transformation and tumor progression, these data suggest a link between *smc3 *haploinsufficiency or loss-of-function and tumorigenesis.

## Methods

### Cells

Human kidney embryonic 293 (HEK293) cells were obtained from ATCC (CRL-1573). p53-null and BAX-null human colon carcinoma HCT116 cells and the parental wild-type cell lines were a kind gift of Dr. Volgelstein. Cells were maintained in DMEM medium supplemented with 10% fetal calf serum.

### Zebrafish breeding

Embryos were obtained from natural mating of wild-type (Oregon, AB) fish and staged according to criteria previously outlined [51].

### Gene cloning

Oligonucleotide sequences matching that of the putative zebrafish *smc3 *gene were retrieved by querying zebrafish dedicated genomic databases using tblastx and the human SMC3 protein sequence (GenBank NM_005445) as query. This protocol enabled to identify a number of overlapping EST clones belonging to a single major transcript and whose translated sequence matched that of the human protein. For cloning of the coding sequence, mRNA isolated from adult male zebrafish was reverse transcribed using Sensiscript reverse transcriptase (Qiagen) priming with oligo-dT. First strand *smc3 *cDNA was then amplified by PCR using *pfu *DNA polymerase and primers of sequence 5'-GCATAAACCGCCATGTACAT TAAA-3' and 5'-TGTGTTTTTCTCTTAGCCGTGAGT-3'. The amplification reaction was extended 10 min by adding *taq *DNA polymerase to generate overhanging termini needed for insertion of the DNA product into the pcDNA3.1-TOPO and the pCRII-TOPO vectors. Capped mRNA for injection in zebrafish embryos was generated using a mMessage mMachine and T7 RNA polymerase kit (Ambion).

### Embryo microinjection

MOs and mRNAs were injected as 1–3 nl bolus into the yolk of 1–2 cell embryos. Post-injection (6 hpf) embryos were sorted, those unfertilized removed and the rest allowed to grow at 28°C. Morpholino-modified antisense oligonucleotides (MO) were obtained from Gene Tools. Those targeting the 5'-UT region of the *smc3 *gene had sequence 5'-GTACATGGCGGTTTATGCACAAAAC-3' and 5'-CTCCTCAGAAACCAAATAAATAAAG-3' respectively. The control 4-base mismatch antisense oligonucleotide had sequence 5'-GTACtTGGCcGTTTtTGCA gAAAAC-3'. p53-MO were generated and used as described in the literature [22]. MO were dissolved in water at a concentration of 4 mM and further diluted in Danieau buffer (58 mM NaCl, 0.7 mM KCl, 0.4 mM MgSO_4_, 0.6 mM Ca(NO_3_)_2_, 5 mM Hepes, pH 7.2) before injection. At selected times the embryo's morphology was examined under a stereomicroscope.

### *In Situ *hybridization of zebrafish embryos

Sense and antisense digoxigenin-labeled riboprobes were generated with T3 or alternatively T7 RNA polymerase using *smc3*-pCRII-TOPO as template. Whole embryo *in situ *hybridization was performed as previously described [52].

### Semi-quantitative RT-PCR and gene expression profiling

mRNA was extracted from 10 embryos using Tri-Reagent and reverse transcribed using oligo-dT primers at 37°C for 2 h. An aliquot of the reaction was used as template for PCR amplification. Reactions were performed in duplicate and limited to 25 cycles to ensure that the amplification was not rate-limited by the available reagents. Products were analyzed on 2% agarose gel. The sets of primers used is listed in Additional file 2.

### Western immunoblot analysis

Embryos were dechorionated and most part of the yolk removed. After washing in PBS, the embryos were collected by centrifugation and resuspended in 100 μl 0.15 M Tris-HCl, pH 7.4 buffer containing 0.1% Tween-20. After homogeneization in an eppendorf tube using a tightly fitted pestel, the embryo debris were removed by centrifugation and the supernatant applied to a 7.5% SDS-PAGE. After separation, the proteins were electroblotted to a nitrocellulose filter and Smc3 detected using goat anti-human SMC3 antibody (Santa Cruz, 1:1000) following the manufacturer directions. The immunocomplexes formed were detected using a Dura kit (Pierce) and the secondary HPR conjugated rabbit anti-goat IgG antibody provided in the kit. For Western immunoblotting of SMC3, 293 human cells were collected in lysis buffer, the proteins separated on 7.5% SDS-PAGE and immunodetected as described for the zebrafish protein. For the normalization of the protein loaded with each sample, SMC3 immunocomplexes-stripped filters were reprobed with anti-human β-actin monoclonal antibody.

### Apoptotic cells detection in embryo whole-mounts

Apoptotic cells were identified in live embryos with Acrydine Orange (AO) or in fixed embryos by *in situ *terminal deoxynucleotidyl transferase (TdT)-mediated dUTP nick-end labeling (TUNEL) reaction. For the AO staining, dechorionated embryos were incubated in 5 μg/ml AO in fish medium (E3) at 28°C for 30 min and washed three timed with 30% Danieau solution for 5 min. Before examination embryos were anaesthetized with 16 mM tricaine. For the TUNEL assay, embryos were collected and washed in PBS before fixation in 4% para-formaldehyde, 0.05% glutaraldehyde, 5 mM EGTA, 5 mM MgSO_4 _(PGEM solution) for 1 hr at 25°C. To facilitate reagent penetration, after washing in PBS, embryos were dehydrated in serial dilution of pure methanol at -20°C for 15 min and then incubated 15 min at RT in 0.1% Triton X-100/0.1% sodium citrate in PBS, followed by three rinse in PBS. An In Situ Cell Death Detection Kit AP (Roche) was used for the assay. After rinsing, embryos were incubated with alkaline phosphatase (AP)-conjugated antifluorescein antibody for 15 min at 37°C and after washing in PBS, the apoptotic cells were visualized by incubation with NBT/BCIP (nitro blue tetrazolium chloride/5-Bromo-4-chloro-3-indolyl-phosphate) reagent. The embryos were then mounted in 50% glycerol in PBS and photographed under a light microscope.

### Phosphohistone H3 labeling

Embryos were fixed overnight in 4% para-formaldehyde in PBS and permeabilized by immersion in -20°C acetone for 10 min. After washing in PBS, the embryos were placed for 30 min in Blocking Reagent (Roche) and incubated overnight at 4°C with 1 μg/ml rabbit anti-pH3 antibody (Santa Cruz). Immunostaining was performed using HRP-conjugated goat anti-rabbit IgG (Pierce) followed by color development in DAB solution. Stained embryos were mounted in methylcellulose and examined under a light microscope.

### Centrosome analysis

Human embryonic kidney 293 cells were transfected with 50 ng/ml of *SMC3*-siRNA. Twenty-four h after transfection the cells were harvested by trypsinization and plated on poly-lysine-coated coverslip. Forty-eight h later, cells were washed with PBS, treated with PGEM containing 0.1% Triton-X100 for 1 hr at 25°C, blocked 1 hr in PBS containing 1% BSA and permeabilized by treatment with methanol at -20°C for 15 min. The fixed cells were then incubated with primary mouse IgG anti-γ-tubulin antibody (1:1000) (Santa Cruz) in blocking buffer at 4° overnight. After reaction with Alexafluor 567-conjugated anti-rabbit IgG (Molecular Probes) and DNA staining with DAPI, the cells were examined under a fluorescence/UV microscope. The acquired γ-tubulin (red) and DNA (blue) signals were superimposed in Photoshop.

### Cell cycle analysis

Batches of 30 embryos were washed twice in ice cold PBS and digested in 5 ml of ice-cold trypsin solution (0.5 μg/ml trypsin in a solution of 0.14 M NaCl, 5 mM KCl, 5 mM glucose, 7 mM NaHCO_3_, 0.7 mM EDTA buffer, pH 7.2) with trituration through a large bore glass pipette for 10 min at room temperature. Cell suspensions were centrifuged at 1000 g for 7 min, resuspendend in 200 μl PBS and fixed in 2 ml cold 70% ethanol at 4°C overnight. The fixed cells were collected by centrifugation, washed in PBS and incubated with propidium iodine (40 μg/ml), and RNAse (10 μg/ml) for 30 min at room temperature. Cell DNA content was analyzed on a Coulter Epic flow cytometer. Human cells were harvested by trypsinization and fixed/stained as described for the zebrafish cells. At the time of acquisition the width (FL2-W) and area (FL2-A) of the PI fluorescence was recorded in linear unit. The ModFitLT software was used to deconvolute DNA histograms according to a four component model which assumes that S phase cells distribute equally between 2 N and 4 N cells and apoptotic cells have DNA content less than 2 N.

## Competing interests

The author(s) declare that they have no competing interests.

## Supplementary Material

Additional File 2 ***Primers used for the semiquantitative analysis of the transcript level of selected zebrafish genes by RT-PCR***. Oligonucleotide sequences matching those of the putative zebrafish genes were retrieved by querying the zebrafish dEST databases with tblastx and the protein sequence of the corresponding human genes. The retrieved zebrafish cDNA sequences were aligned and the longest open reading frame conceptually translated into the putative protein. The cDNA and the polypeptide sequence were in turn used to query the zebrafish genomic database to identify duplicated genes or repeated DNA sequences. Gene-specific PCR primers were designed to generate products of 500–800 bp. Click here for file

Additional File 1 ***Effect of SMC3-siRNA on SMC3 and SMC1 protein level in human HCT116 cells***. A) Western immunoblot assay of SMC3 and SMC1. Wild-type HCT116 cells were transfected with 50 ng/ml *SMC3*-siRNA and harvested 72 h later in lysis buffer. Fifty μg of cell lysate from either untransfected or transfected cells were separated on 7.5% SDS-PAGE and transferred by electroblotting to nitrocellulose filter. SMC3 and SMC1 were then detected using either goat anti-SMC3 or goat anti-SMC1 antibody followed by ECL reaction. Click here for file
