# Supplementary material for: SMC3 knockdown triggers genomic instability and p53-dependent apoptosis in human and zebrafish cells
Source: Mol Cancer. 2006 Nov 2;5:52. doi: 10.1186/1476-4598-5-52 (PMC1636066; doi:10.1186/1476-4598-5-52)
Supplement: Additional File 2 — Primers used for the semiquantitative analysis of the transcript level of selected zebrafish genes by RT-PCR. Oligonucleotide sequences matching those of the putative zebrafish genes were retrieved by querying the zebrafish dEST databases with tblastx and the protein sequence of the corresponding human genes. The retrieved zebrafish cDNA sequences were aligned and the longest open reading frame conceptually translated into the putative protein. The cDNA and the polypeptide sequence were in turn used to query the zebrafish genomic database to identify duplicated genes or repeated DNA sequences. Gene-specific PCR primers were designed to generate products of 500–800 bp. [file 1476-4598-5-52-S2.doc]

***Additional File 1***

*RT-PCR primers*

smc3 forward 5’-CTCTGGATCAGGCCATCAATGACA-3’

reverse 5’-CAGACTGGCTTCCAGACTCTGAAG-3’

smc1 forward 5'-CTCTCTGAACAATCAGACAGTATGGCA-3'

reverse 5'-TTTCCTCAAACAAAGCTGTCCTCTCTT-3'

rad21 forward 5'-GCAATGTTTTACGCCCACTTCGT-3'

reverse 5'-TTCCTACTTCCTCCCTCATGGTG-3'

sa2 forward 5'-CATGCTTTTTGAAGTGGTCAGGCT-3'

reverse 5'-CCAATCATCTTGTTTCTCTCTGCT-3'

p53 forward 5’-TGTCAGCTGGCAAAAACTTG-3'

reverse 5’-ACAAAGGTCCCAGTGGAGTG-3’

mdm2 forward 5’-CAGCAAGGTTGACAACGAGA-3’

reverse 5’-CGAAGGTTGTGTTGGGAGTT-3’

p21 forward 5'-TGAGAACTTACTGGCAGCTTCA-3'

reverse 5'-AGCTGCATTCGTCTCGTAGC-3'

cyclin D1 forward 5'-CTGACTGTCTGCGATCGTGT-3'

reverse 5'-GGGGAGCAGGTACAACGTAA-3'

bax forward 5'-GCAGTGGCAATGACCAGATA-3'

reverse 5'-GGAAAACTCCGACTGTCTGC-3'
