# Supplementary figures and images for: SMC3 knockdown triggers genomic instability and p53-dependent apoptosis in human and zebrafish cells
Source: Mol Cancer. 2006 Nov 2;5:52. doi: 10.1186/1476-4598-5-52 (PMC1636066; doi:10.1186/1476-4598-5-52)

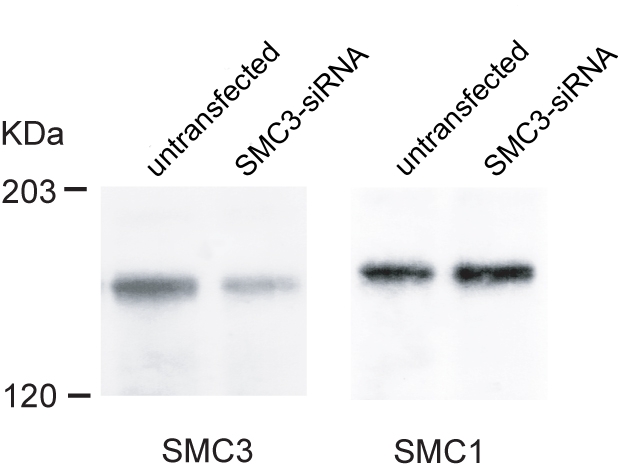

Supplement: Additional File 1 — Effect of SMC3-siRNA on SMC3 and SMC1 protein level in human HCT116 cells. A) Western immunoblot assay of SMC3 and SMC1. Wild-type HCT116 cells were transfected with 50 ng/ml SMC3-siRNA and harvested 72 h later in lysis buffer. Fifty μg of cell lysate from either untransfected or transfected cells were separated on 7.5% SDS-PAGE and transferred by electroblotting to nitrocellulose filter. SMC3 and SMC1 were then detected using either goat anti-SMC3 or goat anti-SMC1 antibody followed by ECL reaction. [file 1476-4598-5-52-S1.jpeg]
